# Supplementary material for: Modeling germline mutations in pineoblastoma uncovers lysosome disruption-based therapy
Source: Nat Commun. 2020 Apr 14;11:1825. doi: 10.1038/s41467-020-15585-2 (PMC7156401; doi:10.1038/s41467-020-15585-2)
Supplement: Supplementary file 1 — Supplementary Information [file 41467_2020_15585_MOESM1_ESM.pdf]

Modeling germline mutations in pineoblastoma uncovers lysosome disruption-based therapy

Chung et al.

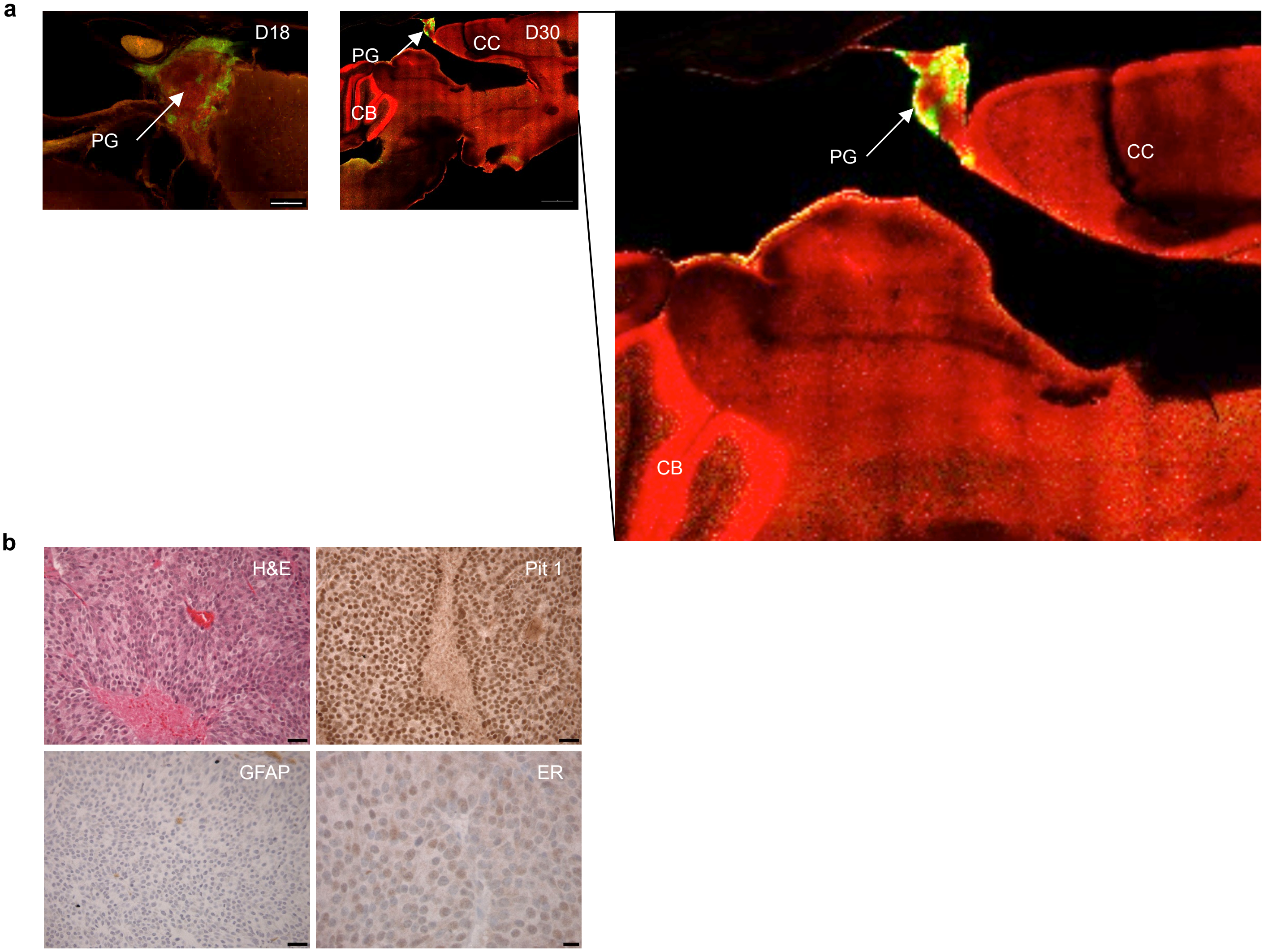

Supplementary Figure 1. WAP-Cre:Rb$^{flox/flox}$:p53$^{flox/flox}$ mice develop pineoblastoma with 100% penetrance as well as occasional pituitary tumors

a. Left, a representative two-photon microscopy image of a pineal gland (PG) with a micro-tumor from 18-day old WAP-Cre:Rb$^{flox/flox}$:p53$^{flox/flox}$:mT/mG mouse. Scale bar, 200 μm. Center, a representative two-photon microscope image of a 30 day old WAP-Cre:Rb$^{flox/flox}$:p53$^{flox/flox}$:mT/mG mouse brain showing a micro-tumor in the pineal gland (right). Scale bar, 1000 μm. CB, cerebellum. Right, enlarged view.

b. Representative H&E and IHC images of a pituitary tumor in a WAP-Cre:Rb$^{flox/flox}$:p53$^{flox/flox}$ mouse. The histology shows small granular cells that stained positive for Pit1 (pituitary transcription factor 1) and ER (Estrogen Receptor) but negative for GFAP. Scale bars, 2 μm.

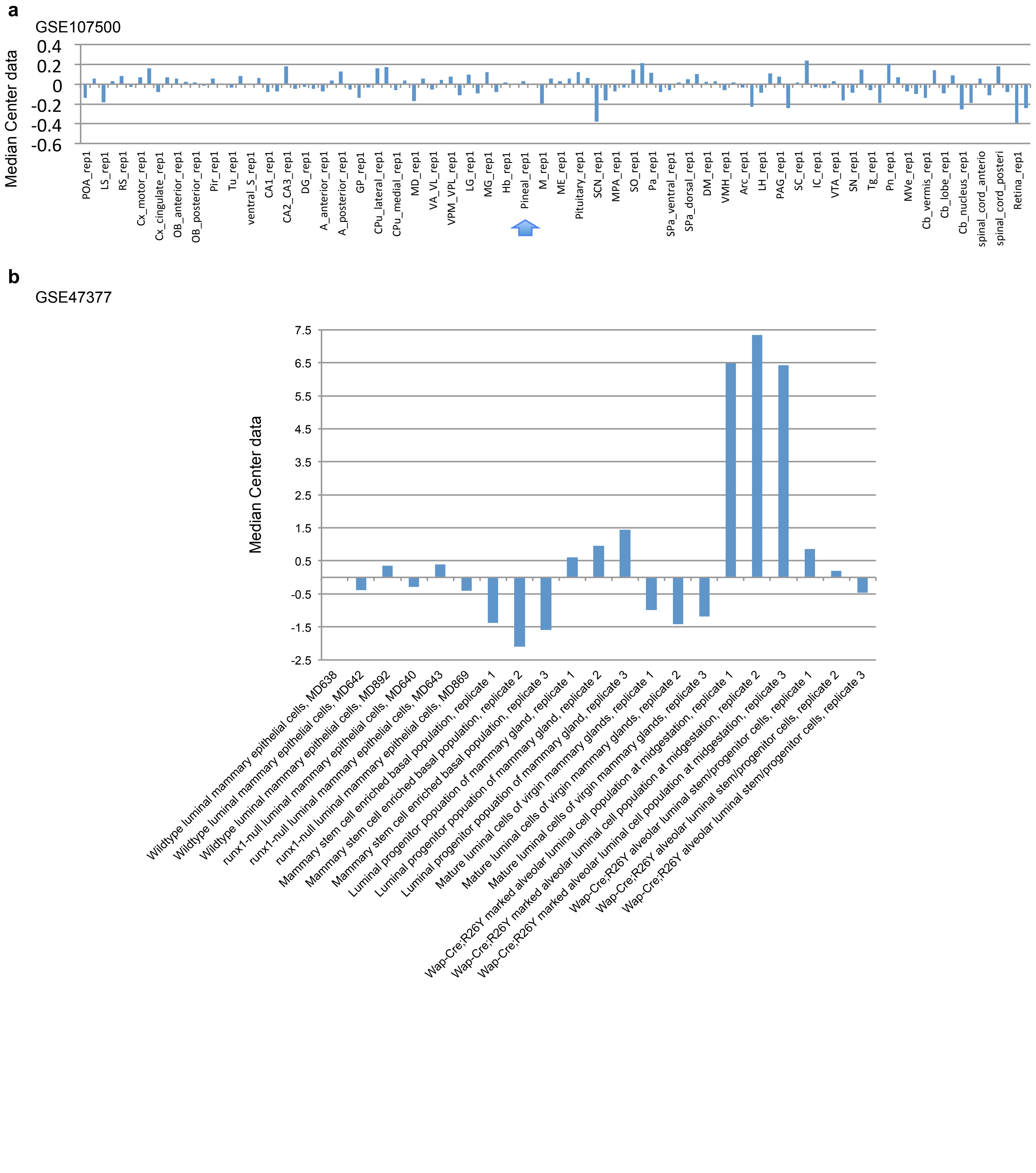

Supplementary Figure 2. Low expression of endogenous Whey Acidic Protein (WAP) gene in the pineal gland

WAP expression in 52 CNS regions of adult mice including pineal gland (**a.**) and mammary epithelial tissues (**b.**). Note high WAP expression in the mammary gland at midgestation and particularly low expression in the pineal gland (arrow) relative to other brain regions.

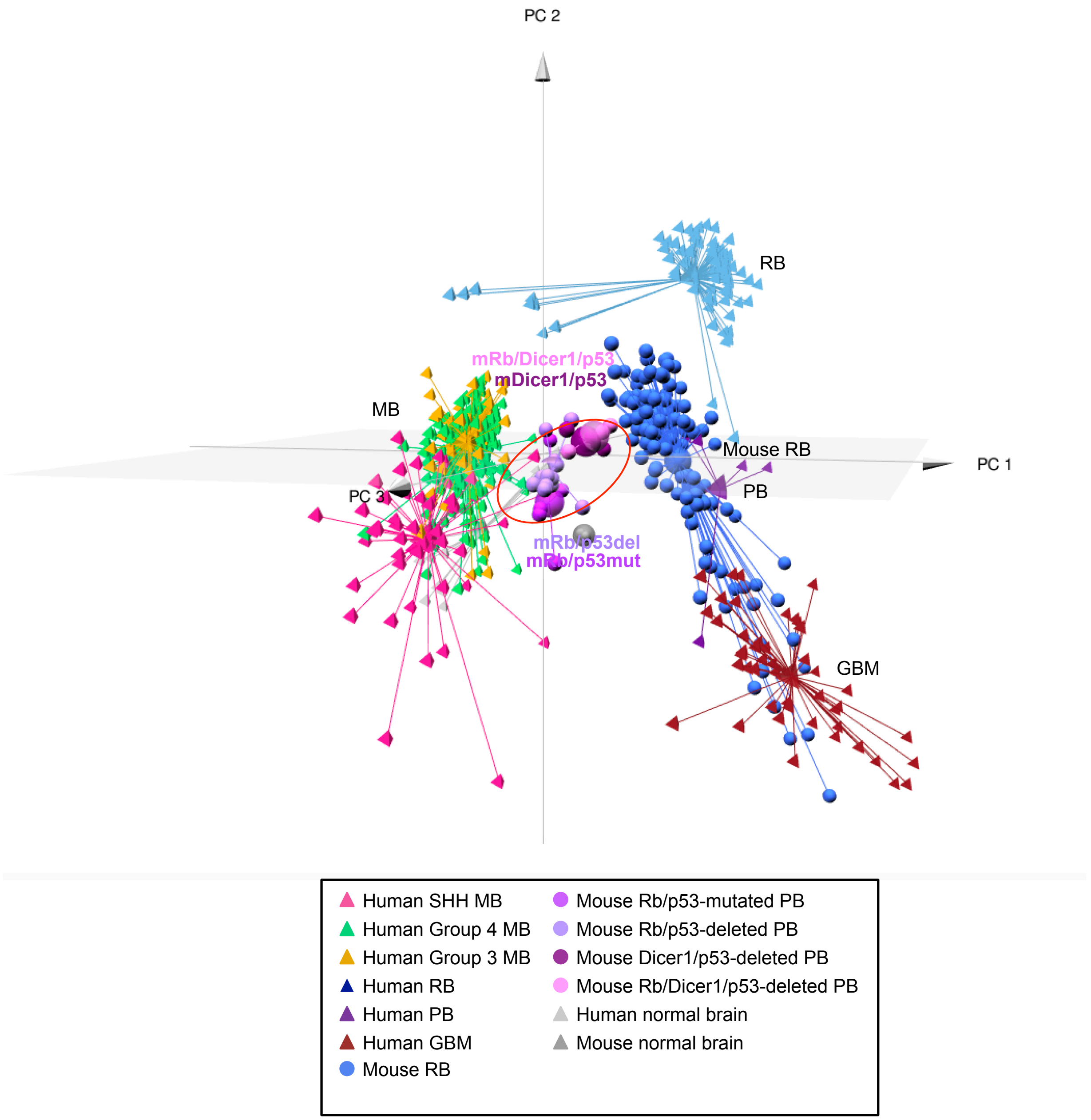

Supplementary Figure 3. A 3D image of GSEA-PC analysis of mouse pineoblastoma models (circled) versus mouse retinoblastoma and human brain tumors. Shown is a 3D image from a slightly different angle than that shown in Fig. 2a.

a

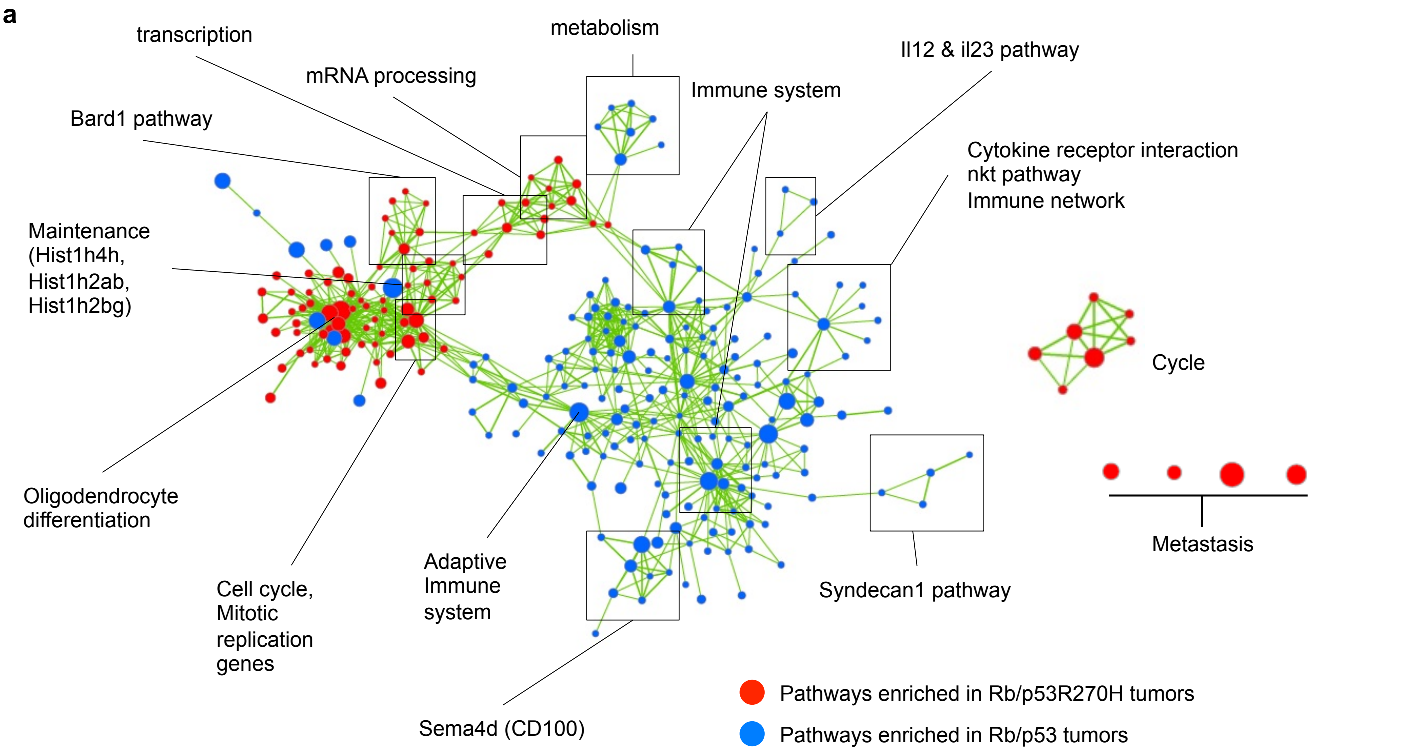

b

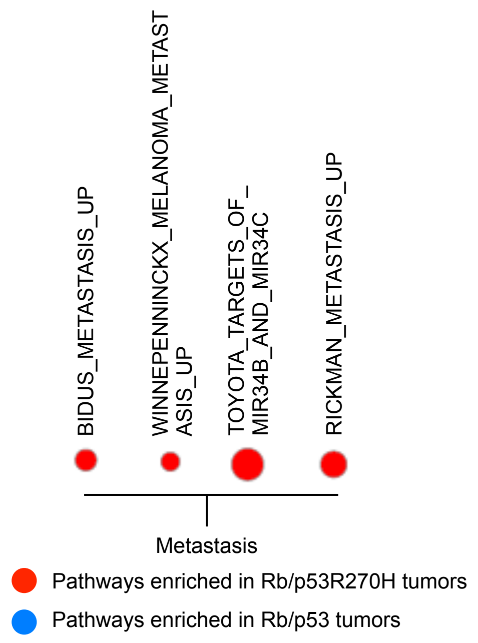

Supplementary Figure 4. Gene set enrichment analysis (GSEA) comparing Rb/p53-deleted and Rb/p53-mutated pineoblastoma

a. Complete GSEA comparing Rb/p53-deleted and Rb/p53-mutated PB shown in part in Fig. 3f. Red dots indicate pathways enriched in Rb/p53-mutated vs Rb/p53-deleted PB; blue dots indicate pathways enriched in Rb/p53-deleted vs Rb/p53-mutated PB.

b. Metastasis pathways enriched in and Rb/p53-mutated compared with Rb/p53-deleted PB.

a

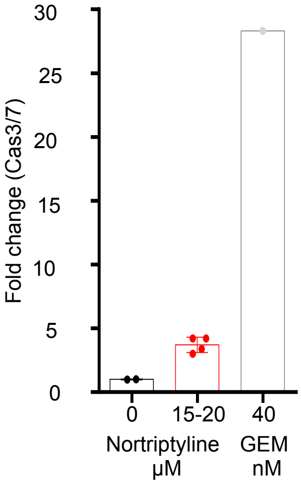

b

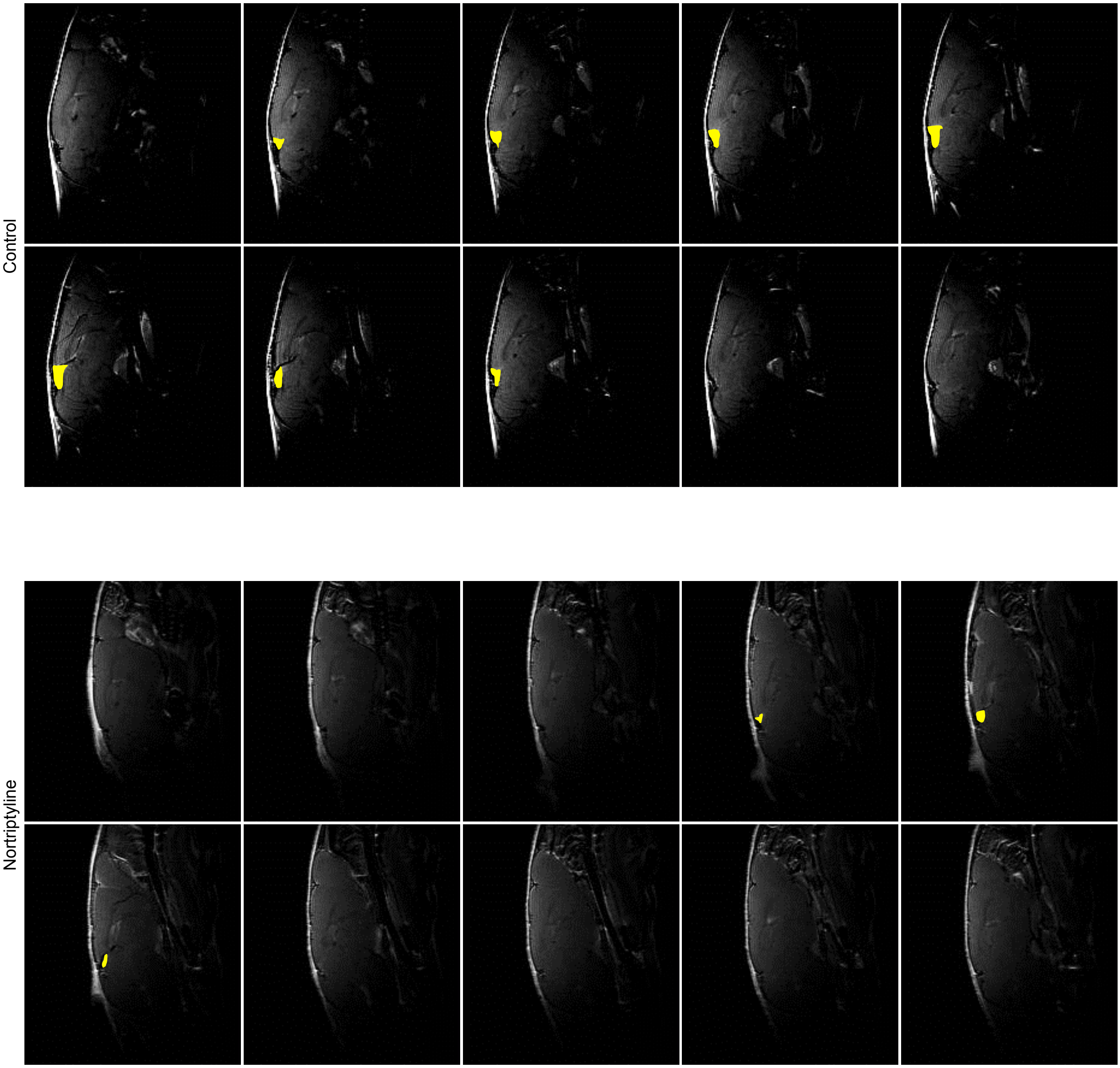

Supplementary Figure. 5

Supplementary Figure 5. Nortriptyline induces primarily a non-apoptotic cell death and suppresses pineoblastoma growth *in vivo*

a. Low caspase3/7 activity in Rb/p53-deficient PB cells (PB-2804) in response to 15-20 μM of nortriptyline, 24 hr post-treatment (n=4). Results are presented as mean ± SD. These assays were performed side by side with those described in Fig. 4d, and the control and Gemcitabine positive arms are the same.

b. An example of serial sagittal MRIs of a WAP-Cre:$Rb^{flox/flox}$:$p53^{flox/flox}$ mouse brain following treatment with vehicle control or nortriptyline (20 mg/kg/d, i.p.) for 5 weeks, used to calculate tumor volume in Fig. 4e. PB areas were digitally marked in yellow. See Source Data files for raw data.

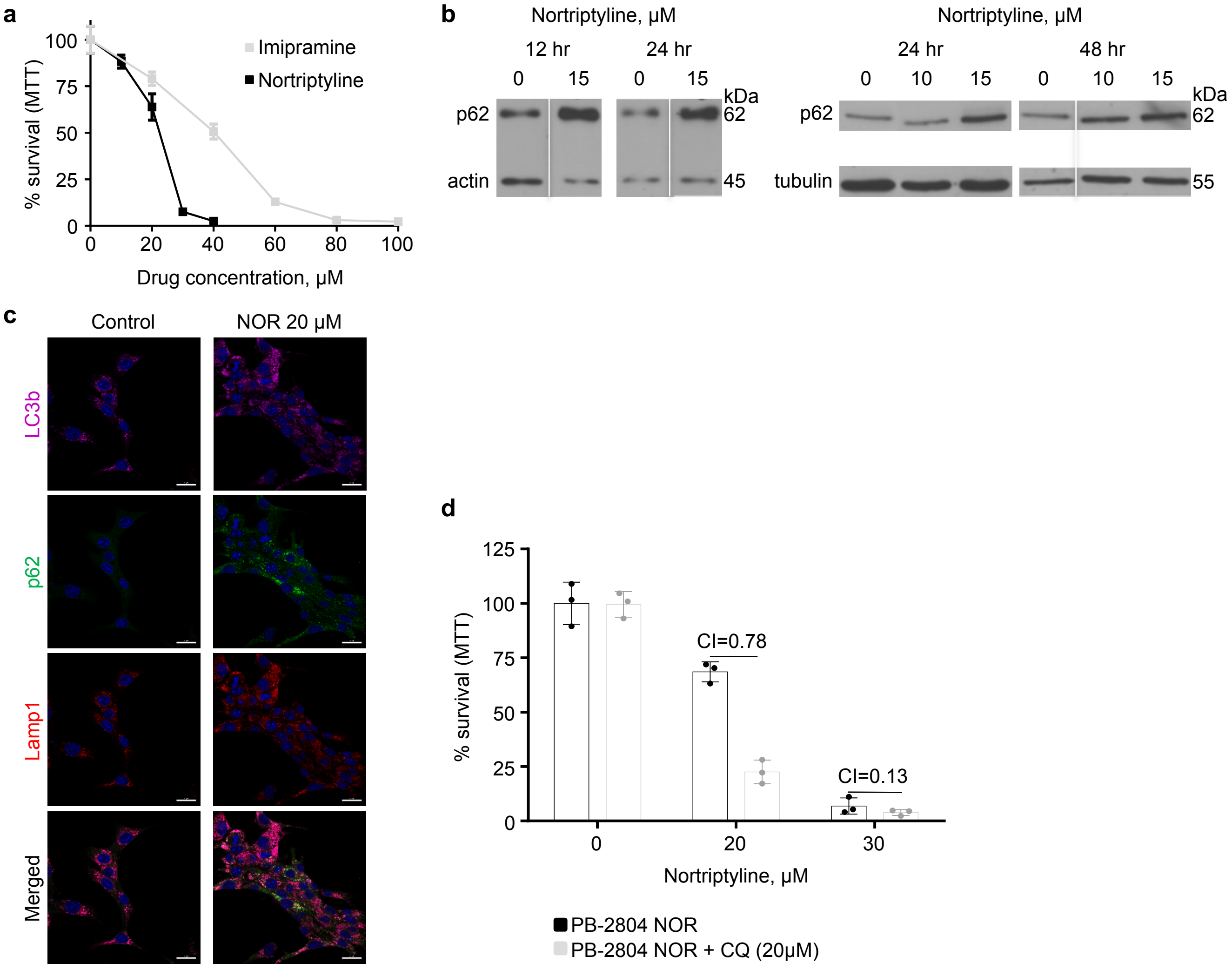

Supplementary Figure 6. Nortriptyline impedes autophagic flux and synergises with chloroquine to restrict growth

a. MTT assays of mouse PB cells treated for 24 hr with increasing concentrations of nortriptyline (n=3) or imipramine (n=3).

b. Immunoblots for p62 in mouse PB cells treated with vehicle control or nortriptyline for indicated time periods and concentrations.

c. Representative IF for LC3b (purple), p62 (green) and lamp1 (red) in PB cells treated with vehicle control or nortriptyline for 2 hr. Scale bar, 20 μm.

d. MTT assay of mouse PB cells treated with vehicle control (n=3), chloroquine alone (n=3), increasing concentrations of nortriptyline alone (n=3) or together with chloroquine (n=3; 20 μM). Cooperation Index was calculated using CompuSyn (CI<0.85 denotes synergy). Results are presented as mean ± SD in **a** and **d**. See Source Data files for raw data.

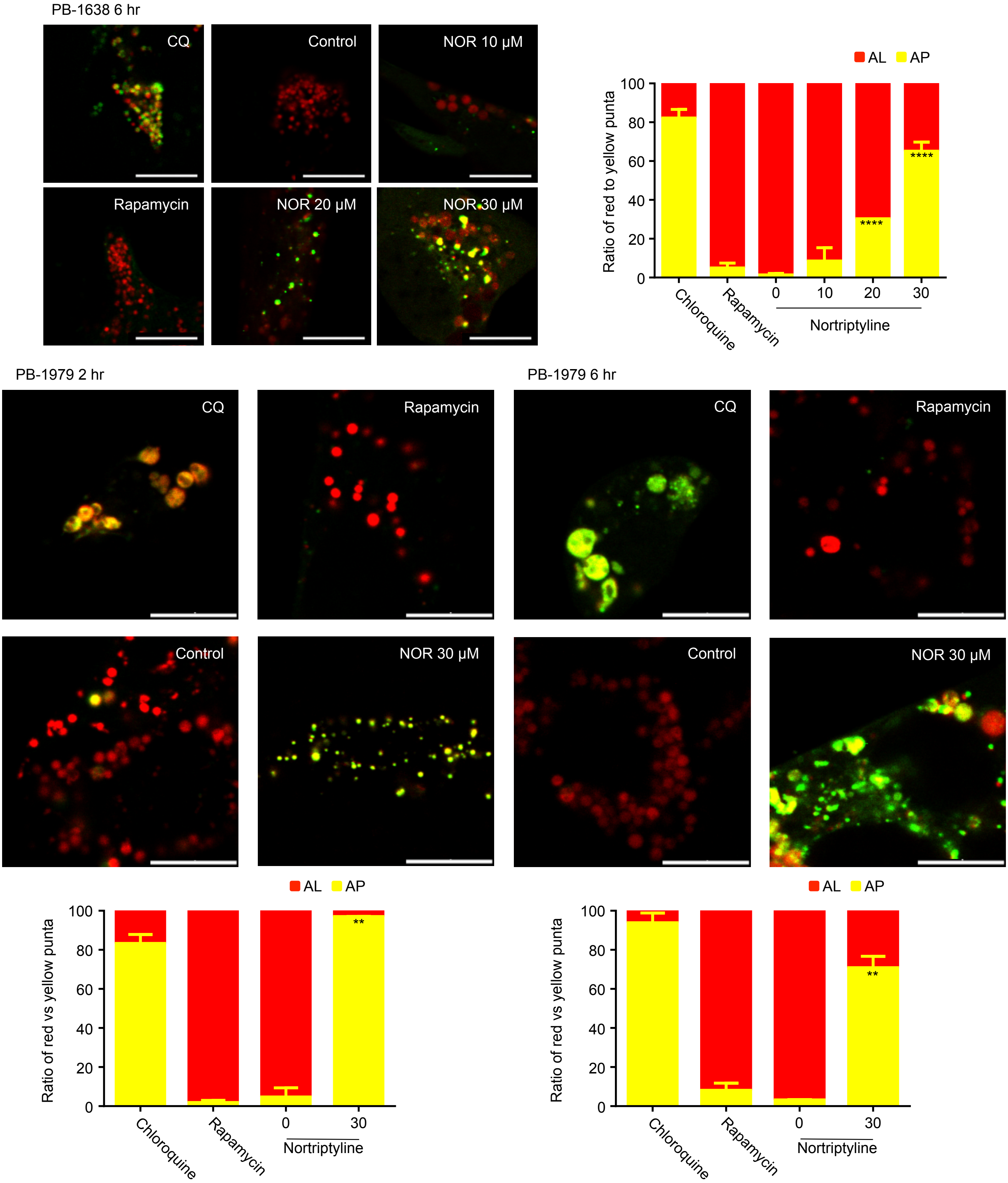

Supplementary Figure. 7

Supplementary Figure 7. Nortriptyline induces accumulation of autophagosomes or non-functional autolysosomes

Analysis of autophagosomes (AP, yellow) and autolysosomes (AL, red) using mRFP-GFP-LC3 reporter plasmid transfected into Rb/p53-deficient PB-1638 or PB-1979 cells. Cells were treated with control vehicle, chloroquine (CQ, 20 μM), rapamycin (500 nM), or increasing concentration of nortriptyline for 2 hr or 6 hr as indicated. Intensities of yellow and red puncta were measured using ImageJ with JACoP plug-in. Scale bar, 10 μm. Two-tailed Student's t-test, *p<0.05, **p<0.01 and ****p<0.0001. Results are presented as mean ± SD. See Source Data files for raw data.

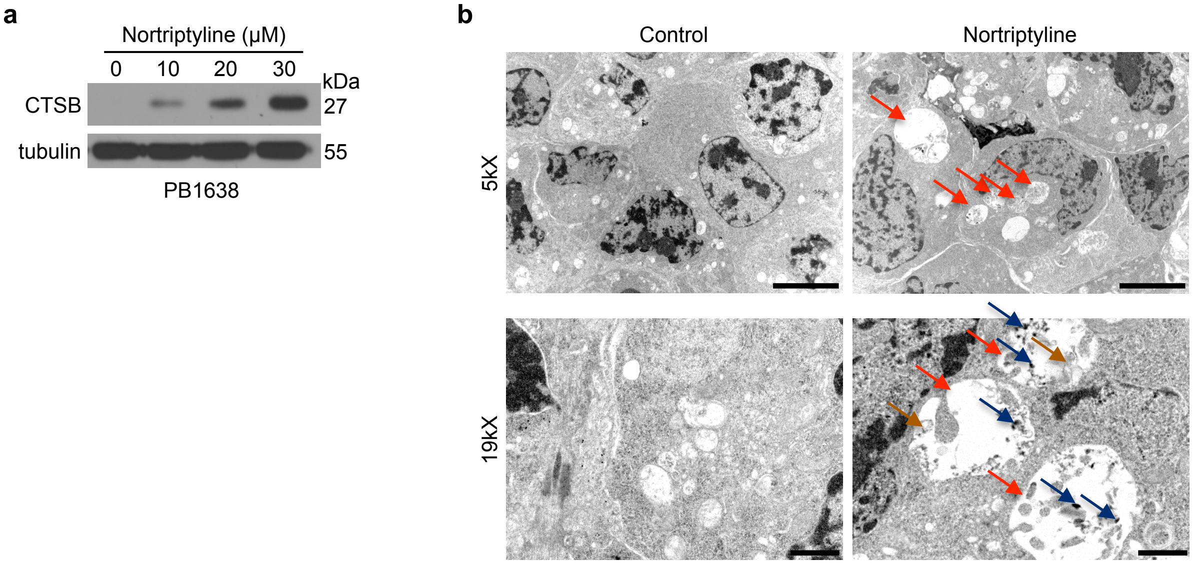

Supplementary Figure 8. Nortriptyline induces cathepsin B release and impairs autophagic flux in pineoblastoma cells

a. Immunoblot analysis of cathepsin B expression in cytoplasmic fractions of PB1638 cells treated with vehicle control or increasing concentrations of nortriptyline for 2 hrs.

b. Representative TEM images of PB-2804 cells treated with vehicle control or 30μM nortriptyline for 2 hr. Red arrows, autophagic vacuoles; blue arrow, lipid droplets; brown arrow, partially degraded contents. Scale bars, 5μm (top panels) and 1μm (bottom panels). For raw data see Source Data files.

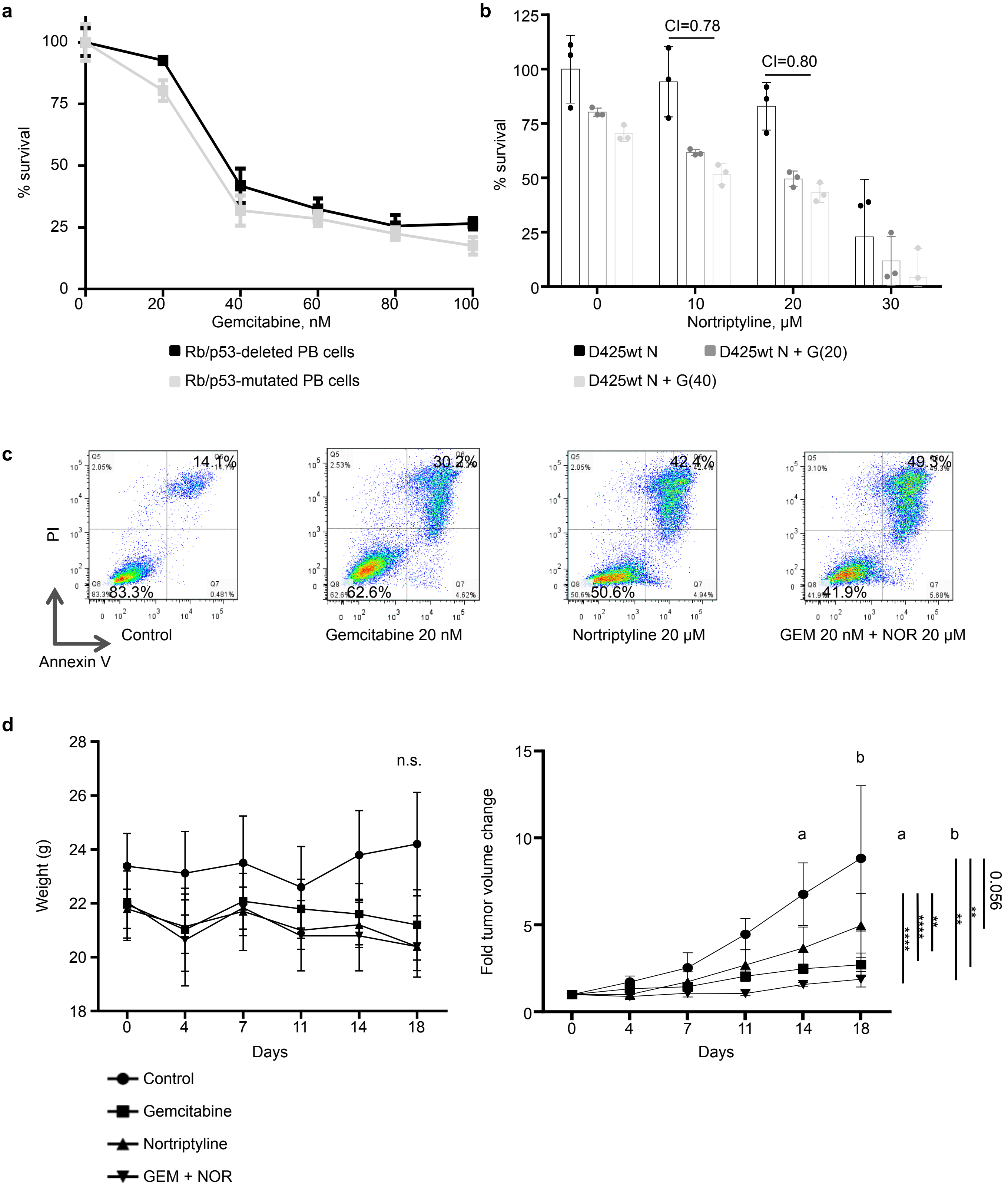

Supplementary Figure. 9

Supplementary Figure 9. Nortriptyline synergises with gemcitabine to suppress pineoblastoma growth

a. MTT assays of Rb/p53-deleted PB (n=3; PB-1638, black) and Rb/p53-mutated PB (n=3; PB-50, grey) cells treated for 24 hr with vehicle control or increasing concentrations of gemcitabine.

b. MTT assay of human group 3 medulloblastoma D425wt cells treated with vehicle control (n=3), gemcitabine alone (n=3), nortriptyline alone (n=3) or both drugs (n=3). Cooperation Index was calculated using CompuSyn (CI<0.85 denotes synergy).

c. Annexin V/PI flow cytometry analysis of mouse PB-1638 cells treated with control vehicle, gemcitabine (20 nM), nortriptyline (20 μM), or both drugs, showing reduced viability and increased necrotic/late apoptotic (AnnexinV+/PI+) cells following NOR plus GEM treatment.

d. Left, weight of PB-bearing NOD/SCID mice treated with control (n=5), gemcitabine (n=5), nortriptyline (n=4), or combination of gemcitabine plus nortriptyline (n=5). Right, fold tumor volume change in NOD/SCID mice injected *sub. cu.* with mouse PB cells and treated with control, gemcitabine, nortriptyline, or combination of gemcitabine plus nortriptyline. Two-tailed Student's t-test, **p<0.01, ****p<0.0001. Results are presented as mean ± SD in **a**, **b** and **d**. See Source Data files for raw data.
