# Supplementary material for: Modeling germline mutations in pineoblastoma uncovers lysosome disruption-based therapy
Source: Nat Commun. 2020 Apr 14;11:1825. doi: 10.1038/s41467-020-15585-2 (PMC7156401; doi:10.1038/s41467-020-15585-2)
Supplement: Supplementary file 3 — Reporting Summary [file 41467_2020_15585_MOESM3_ESM.pdf]

nature research

Corresponding author(s): Eldad Zacksenhaus
Last updated by author(s): Mar 9, 2020

Reporting Summary

Nature Research wishes to improve the reproducibility of the work that we publish. This form provides structure for consistency and transparency in reporting. For further information on Nature Research policies, see Authors & Referees and the Editorial Policy Checklist .

Statistics

For all statistical analyses, confirm that the following items are present in the figure legend, table legend, main text, or Methods section.

n/a	Confirmed	
☐	☒	The exact sample size (*n*) for each experimental group/condition, given as a discrete number and unit of measurement
☐	☒	A statement on whether measurements were taken from distinct samples or whether the same sample was measured repeatedly
☐	☒	The statistical test(s) used AND whether they are one- or two-sided<br>*Only common tests should be described solely by name; describe more complex techniques in the Methods section.*
☒	☐	A description of all covariates tested
☒	☐	A description of any assumptions or corrections, such as tests of normality and adjustment for multiple comparisons
☐	☒	A full description of the statistical parameters including central tendency (e.g. means) or other basic estimates (e.g. regression coefficient) AND variation (e.g. standard deviation) or associated estimates of uncertainty (e.g. confidence intervals)
☐	☒	For null hypothesis testing, the test statistic (e.g. *F*, *t*, *r*) with confidence intervals, effect sizes, degrees of freedom and *P* value noted<br>*Give P values as exact values whenever suitable.*
☒	☐	For Bayesian analysis, information on the choice of priors and Markov chain Monte Carlo settings
☒	☐	For hierarchical and complex designs, identification of the appropriate level for tests and full reporting of outcomes
☒	☐	Estimates of effect sizes (e.g. Cohen's *d*, Pearson's *r*), indicating how they were calculated

Our web collection on statistics for biologists contains articles on many of the points above.

Software and code

Policy information about availability of computer code

Data collection	No software was used to collect data
Data analysis	CompuSyn, MIPAV, ImageJ (1.52a), Microsoft Office 365, GraphPad Prism 6 (6.01), GSVA package (version 1.32.0) in R(3.5.0)

For manuscripts utilizing custom algorithms or software that are central to the research but not yet described in published literature, software must be made available to editors/reviewers. We strongly encourage code deposition in a community repository (e.g. GitHub). See the Nature Research guidelines for submitting code & software for further information.

Data

Policy information about availability of data

All manuscripts must include a data availability statement. This statement should provide the following information, where applicable:
- Accession codes, unique identifiers, or web links for publicly available datasets
- A list of figures that have associated raw data
- A description of any restrictions on data availability

For figures 2c and d, human SHH MB and PB data were obtained from https://hgserver1.amc.nl/cgi-bin/r2/main.cgi - Mixed Pediatrix PDX (public)- Olson-55-MAS5.0-u133p2.
All relevant data are available from the corresponding author upon reasonable request. Source data for figures are provided as Source Data file.
All of our microarray data are deposited with accession number GSE124537. GSE37382, GSE36245, GSE29685, GSE24673 and GSE59983 are available publicly

Field-specific reporting

Please select the one below that is the best fit for your research. If you are not sure, read the appropriate sections before making your selection.

Life sciences study design

All studies must disclose on these points even when the disclosure is negative.

Sample size	In general, experiments were done in 3 biological replicates, each in triplicates, and statistical significance was calculated as indicated. Sample size of 3 are routinely used in this field.
Data exclusions	No data were excluded from the analyses
Replication	Experiments were done 3 times when possible to verify the reproducibility of the experimental findings. There are no findings that were not replicated. Results were successfully replicated in three independent attempts
Randomization	For all in vivo drug treatment experiments, mice were randomized after initial tumor measurement. ANOVA was performed to test for differences in tumor size in different groups
Blinding	Blinding was not possible as the same person performed and analyzed experiments

Reporting for specific materials, systems and methods

We require information from authors about some types of materials, experimental systems and methods used in many studies. Here, indicate whether each material, system or method listed is relevant to your study. If you are not sure if a list item applies to your research, read the appropriate section before selecting a response.

Materials & experimental systems

n/a	Involved in the study	
☐	☒	Antibodies
☐	☒	Eukaryotic cell lines
☒	☐	Palaeontology
☐	☒	Animals and other organisms
☒	☐	Human research participants
☒	☐	Clinical data

Methods

n/a	Involved in the study	
☒	☐	ChIP-seq
☐	☒	Flow cytometry
☐	☒	MRI-based neuroimaging

Antibodies

Antibodies used	(in order of Supplier name, Cat#, Lot# where applicable) p53 (Santa Cruz, #SC-126, F3017). synaptophysin (Cell Signaling, #36406). cathepsin B (1:800, Cell Signaling, #31718, #1), GFAP (Cell Signaling, #3670, #3), Lamp-1 (DSHB, 1D4B), LC3B (Cell Signaling, #3868, #11), ki67 (Biocare Medical, #CRM325), p62 (Abnova, #H00008878-M01, G1251-2C11), phospho-Rb (Cell Signaling, #9301, #11), α/β-tubulin (Cell Signaling, #2148, #7), actin (DSHB, JLA20), OX42 (DSHB, M1), Alexa Fluor 488 anti-mouse (1:200, Life Technology, #Z25002), Alexa Fluor 488 anti-rabbit (1:200, Life Technology, #Z25302), Cy5 anti-rat (1:200; Life Technology, #A10525), Alexa Fluor 568 anti-mouse (1:200, Life Technology, #Z25006), Alexa Fluor 568 anti-rabbit (1:200, Life Technology, #Z25306), Biotinylated Goat Anti-Rabbit (1:200, Vector Laboratories, #BA-1000), Biotinylated Horse Anti-Mouse (1:200, Vector Laboratories, BA-2000), nestin (1:100, Abcam, #ab11306), neurofilament (5 μg/ml, DSHB, #2H3), PAX6 (5 μg/ml, DSHB, #PAX6), 5-HT (1:1000, ImmunoStar, #20080).
Validation	For antibodies listed above, validations and citations can be found on the manufacturer's website.

Eukaryotic cell lines

Policy information about cell lines

Cell line source(s)	D425wt - donated by Dr. Michael D. Taylor, Canada. TS13-19 - provided by Dr. Seok-Gu Kang, Republic of Korea. HaCaT - by Dr. Anton Neschadim, Canada.
Authentication	None of the cell lines were authenticated
Mycoplasma contamination	Cell lines were not tested for mycoplasma contamination
Commonly misidentified lines (See ICLAC register)	Did not use any commonly misidentified lines

Animals and other organisms

Policy information about studies involving animals; ARRIVE guidelines recommended for reporting animal research

Laboratory animals	Mus musculus (mixed background, males and females, 0-6 months; NOD/SCID mouse, males, 4-6 weeks)<br>Mice were group-housed (5 per cage) in a temperature- and humidity- controlled room on a 12h light/dark cycle with access to food and water
Wild animals	Study did not involve wild animals.
Field-collected samples	Study did not involve samples collected from the field.
Ethics oversight	Research Institute Animal Research Commeittee at University Health Network, Canada.

Note that full information on the approval of the study protocol must also be provided in the manuscript.

Flow Cytometry

Plots

Confirm that:

- [x] The axis labels state the marker and fluorochrome used (e.g. CD4-FITC).
- [x] The axis scales are clearly visible. Include numbers along axes only for bottom left plot of group (a 'group' is an analysis of identical markers).
- [x] All plots are contour plots with outliers or pseudocolor plots.
- [x] A numerical value for number of cells or percentage (with statistics) is provided.

Methodology

Sample preparation	Samples were treated with vehicle control or drugs. Cells were stained with PI and FITC-Annexin V
Instrument	BD Fortessa analytical instrument
Software	FlowJo7.6
Cell population abundance	Cell populaition can be seen in the bottom of sample name box.Cell numbers in all samples are more than 10,000.
Gating strategy	The boundaries between positive and negative staining cell populations are defined by one sample's single staining ,repectively.

- [x] Tick this box to confirm that a figure exemplifying the gating strategy is provided in the Supplementary Information.

Magnetic resonance imaging

Experimental design

Design type	Studying the effect of drugs on tumor growth
Design specifications	Experimental mice were imaged twice (5 weeks interval)
Behavioral performance measures	Experiments did not require behavioral performance measurement

Acquisition

Imaging type(s)	Structural
Field strength	7 Tesla
Sequence & imaging parameters	Mice were oriented in prone position on a dedicated slider bed and RF coil configuration for mouse brain imaging. Tumors were visualized using a multislice 2D sagittal contrast-enhanced T1-weighted RARE technique (TE/TR = 10/1000 ms; echo train length 2; 200 x 180 matrix with 100 um in-plane resolution for 20 x 18 mm field of-view; at least 19 slices of 300 um thickness centred on the mid-line of the brain; 8 averages; 12 min acquisition time).
Area of acquisition	The brain was scanned sagittally as described above to cover most of the mouse brain. Pineoblastomas are found in the mid-line of the mouse brain where the pineal gland is located. The region was chosen as a starting point to scan the entire tumor mass.

Diffusion MRI [ ] Used [x] Not used

Preprocessing

Preprocessing software	MRIs were viewed using Medical Image Processing, Analysis & Visualization (MIPAV) - v7.3.0 software
Normalization	Data were not normalized
Normalization template	Data were not normalized
Noise and artifact removal	Was not required to perform these steps
Volume censoring	Did not perform volume censoring

Statistical modeling & inference

Model type and settings	MRI was done to measure the tumor volume.
Effect(s) tested	Mice were not subject to any tasks or stimuli

Specify type of analysis: ☐ Whole brain ☒ ROI-based ☐ Both

Anatomical location(s): Mid-line of the brain - this is where the pineal gland is located and where tumor sare form

Statistic type for inference (See Eklund et al. 2016)	No statistics was done for MRI. Statistic analysis was applied for differences in tumor volume as indicated.
Correction	No correction was required

Models & analysis

n/a	Involved in the study
☒	☐ Functional and/or effective connectivity
☒	☐ Graph analysis
☒	☐ Multivariate modeling or predictive analysis
